# The impact of the integrated labor-delivery-recovery-postpartum unit on maternal–neonatal outcomes and psychological experiences among low-risk parturients: a prospective cohort study from a high-volume tertiary center in china

**DOI:** 10.3389/fmed.2026.1845643

**Published:** 2026-06-22

**Authors:** Hui Zheng, Meihong Zhu, Lili Xu, Lei Zhou, Yun Xu, Luqin Huang, Yali Guan, Ying Liang, Yi Gao, Zekai Yu, Qi Chen, Shixia Xu, Min Chen, Zhicheng Xu, Xiaotian Wang, Caitao Ying, Kai Chen, Hailing Hong, Wei Xiong

**Affiliations:** 1 Department of Obstetrics, Shangrao Maternal and Child Health Hospital, Shangrao, China; 2 Nursing School of the First Affiliated Hospital of Nanchang University, Nanchang, China; 3 Nanchuan Hospital of Traditional Chinese Medicine, Chongqing, China; 4 School of Computer Science and Technology, Hangzhou Dianzi University, Hangzhou, China; 5 Department of Neonatology, Neonatal Intensive Care Unit, Shangrao Maternal and Child Health Hospital, Shangrao Children’s Hospital, Shangrao, China; 6 Shangrao Maternal and Child Health Hosptal, Shangrao, China

**Keywords:** Bayesian, childbirth, LDRP, neonatal transfer, postpartum hemorrhage

## Abstract

**Objective:**

To evaluate the clinical effectiveness of the integrated labor-delivery-recovery-postpartum (LDRP) model among low-risk parturients at a tertiary hospital in China.

**Methods:**

A total of 542 low-risk, full-term, singleton parturients voluntarily selected either the LDRP or traditional delivery mode. Outcomes between groups were compared using propensity score matching, multivariate regression, Bayesian analysis, and machine learning.

**Results:**

The LDRP group had shorter labor duration (7.3 h vs. 9.8 h), and significantly lower cesarean section rates (1.2% vs. 4.6%) and neonatal transfer rates (5.4% vs. 16.0%; all *P* < 0.05). Postpartum hemorrhage rates were also lower in the LDRP group (1.2% vs. 6.0%), but this difference was not statistically significant after multivariate adjustment (*P* = 0.235). Women in the LDRP group more frequently reported feeling “calm and relaxed” (17.7% vs. 0.4%) but had higher latent-phase pain scores (2.0 vs. 1.0). Bayesian analysis confirmed that LDRP independently reduced neonatal transfer risk, though its protective effect against postpartum hemorrhage was insufficiently robust.

**Conclusion:**

The LDRP model effectively shortens labor, reduces cesarean section and neonatal transfer rates, and improves maternal psychological experience. Its independent protective effect on postpartum hemorrhage requires further validation.

## 1 Introduction

Childbirth is not merely a physiological event but a profound psychological and social experience. In China, where the one-child policy shaped obstetric practices for decades and where birth volumes in tertiary hospitals are among the highest globally, the birthing environment has traditionally prioritized efficiency and medical safety over continuity and maternal experience. The conventional model, separating labor, delivery, and postpartum recovery into distinct functional units, requires parturients to transfer between rooms multiple times. This fragmented approach, while logistically efficient, may disrupt the continuity of care, heighten maternal anxiety due to environmental unfamiliarity, and potentially compromise labor progression and subjective experience (1).

To date, evidence on the labor-delivery-recovery-postpartum (LDRP) model comes predominantly from high-income countries with low birth rates, well-staffed maternity units, and established family-centered care traditions (2–5). In these settings, LDRP has been associated with improved maternal satisfaction and reduced cesarean rates. However, whether these findings translate to high-volume, resource-constrained, and culturally distinct settings, such as China’s tertiary maternity centers, remains unknown. In the Chinese context, where family involvement is deeply valued but hospital infrastructure often prioritizes throughput, the feasibility, safety, and effectiveness of implementing a continuous, rooming-in LDRP model have not been systematically evaluated. Despite its theoretical benefits, the global implementation of the LDRP model remains highly uneven, with limited high-quality evidence. Current studies evaluating LDRP outcomes are predominantly from high-income countries and frequently involve descriptive analyses or non-randomized controlled trials. These studies typically emphasize maternal satisfaction, cost-effectiveness, or rates of specific interventions (e.g., analgesia) (1, 6, 7). For low-risk parturients, systematic and comprehensive comparative analyses of LDRP’s impact on objective clinical outcomes (e.g., labor duration, perineal trauma, postpartum hemorrhage, neonatal outcomes) and dynamic psychological and pain indicators during labor are still lacking.

However, methodological limitations remain in the current literature. First, selection bias frequently emerges as a concern: since the choice of birthing model often reflects personal preferences, cultural norms, or differential access to information, true randomization is seldom feasible. Additionally, many observational studies insufficiently adjust for essential confounders, such as baseline maternal characteristics and obstetric variables, which may obscure the actual effects of the LDRP model. Equally important, there is limited investigation into how psychological and physiological indicators dynamically interact throughout labor. Collectively, these shortcomings have hindered clarification of the specific benefits and underlying mechanisms of LDRP care, thereby restricting evidence-based adoption, broader clinical implementation, and policy promotion.

Given this context, a prospective cohort study was conducted at our medical center to systematically compare the labor process, maternal and neonatal outcomes, and perinatal psychological experiences in low-risk, full-term parturients who voluntarily chose either the LDRP model or a conventional delivery room setting. By adjusting for key confounding factors, we aimed to better identify the independent effects of the LDRP model and preliminarily examine potential mediating pathways, for instance, whether improvements in emotional state might correspondingly influence pain perception and stress responsiveness.

This study therefore represents the first prospective cohort evaluation of the LDRP model in a high-volume Chinese tertiary maternity center. By systematically comparing labor processes, maternal-neonatal outcomes, and dynamic pain-emotion interactions between women who voluntarily chose LDRP versus traditional delivery care, we aim to provide empirical evidence from a non-Western, high-birth-volume setting. These findings will inform whether the LDRP model—developed and validated in low-birth-rate Western contexts—can be effectively adapted to improve perinatal care quality in China and similar settings.

## 2 Materials and methods

This study adopted a single-center, prospective, observational cohort design. Quantitative methods were used to systematically compare clinical and psychological outcomes among low-risk parturients who autonomously selected their birthing model, specifically focusing on differences between the LDRP and traditional delivery room models. A prospective cohort design was selected because it ethically respects maternal autonomy and effectively allows observation and comparison of associations between a natural exposure (birthing model) and multiple outcomes, reflecting real clinical conditions.

### 2.1 Standardized data collection process

All data were obtained from our hospital’s obstetric center (Jan 2025–Jan 2026). During admission, midwives provided a detailed introduction to the environmental and service differences between the LDRP model and standard delivery rooms. Parturients then made voluntary, informed decisions, mirroring real clinical decision-making, although this introduced potential selection bias. To address this issue, comprehensive baseline data were prospectively collected for subsequent adjustment.

#### 2.1.1 Inclusion and exclusion criteria

To ensure a relatively homogeneous low-risk cohort, strict inclusion criteria were applied: participants had to have a singleton pregnancy with cephalic presentation, be at term (≥ 37 weeks’ gestation), and have “no risk factors” based on the national Standard for Maternal Pregnancy Risk Screening and Assessment Management (8–15). Individuals diagnosed with pregnancy complications, comorbidities, or those with incomplete medical records were excluded. These measures aimed to enhance comparability across essential pathophysiological characteristics, thus ensuring that observed differences could be more confidently attributed to the birthing model rather than underlying maternal or pregnancy-related factors.

#### 2.1.2 Neonatal transfer criteria and ward setting description

In this study, the neonatal transfer rate refers to the proportion of neonates transferred to the neonatal department for additional medical intervention after birth. Notably, our neonatal department is a comprehensive ward, internally differentiated into a neonatal intensive care unit (NICU) and a general neonatal ward. Neonates requiring separation from rooming-in for further medical monitoring or treatment are admitted to this integrated ward. Thus, “NICU transfer” in this study is an administrative indicator covering a broad range of conditions, from severe illnesses to common neonatal conditions requiring short-term observation or treatment.

Specific transfer criteria include, but are not limited to: (1) neonatal jaundice requiring phototherapy; (2) suspected or confirmed neonatal pneumonia or infections requiring intravenous antibiotic therapy; (3) feeding intolerance, vomiting, or excessive weight loss needing medical intervention; (4) observation required for low birth weight or small-for-gestational-age infants; (5) intrapartum asphyxia or respiratory distress requiring respiratory support or close monitoring. This setup accurately reflects clinical practices in our center, ensuring that the outcome indicator (transfer rate) sensitively captures differences in overall medical resource utilization between the two neonatal groups, rather than merely severe complications. For consistency and clarity, subsequent charts and discussions uniformly refer to this indicator as NICU Transfer (16, 17).

### 2.2 Objective clinical data

Clinical data were directly extracted from the electronic medical record system. These included demographic characteristics, labor parameters, mode of delivery, postpartum blood loss (measured using volumetric and gravimetric methods), neonatal indicators, and umbilical cord arterial blood gas analysis results. All tests were performed by the hospital laboratory according to standard operating procedures. *Subjective psychological and pain data*: Uniformly trained nurses (not the responsible midwife) assessed these data on-site, using standardized scales during the latent phase, active phase, and expulsion stage of labor. Emotional states were assessed based on the parturients’ subjective feelings. Pain intensity was measured using the internationally recognized Numeric Rating Scale (NRS, 0–10) (18, 19). Independent nurse assessors were utilized to minimize potential bias caused by the presence of the responsible midwife. All case report forms were completed and entered within 24 h postpartum and underwent 100% verification by a second researcher to ensure data completeness and accuracy.

### 2.3 Statistical analysis strategy and rationale

This study employed a multi-dimensional analytical approach, integrating three complementary methods: Frequentist multivariate logistic regression as the primary analysis to estimate the adjusted independent effect (odds ratios) of LDRP; Bayesian logistic regression to quantify uncertainty in effect estimates, particularly suitable for rare outcomes; and machine learning as a supplementary tool to explore nonlinear relationships and validate the predictive importance of LDRP, without inferring causality. Standard statistical procedures were used for categorical variables to ensure analytical rigor. Thus, continuous variables are presented as median (interquartile range) and were compared using the Mann-Whitney U test. Categorical variables are summarized as counts and percentages, and were analyzed using the Chi-square test or Fisher’s exact test (when expected counts were below five).

To control for baseline imbalances affecting outcomes, multivariate logistic regression models were constructed. Initially, a “core model,” adjusting only for core obstetric variables (age, parity, gravidity, gestational age), was built. Subsequently, a “full model” was developed by including potential mediators or confounders (e.g., analgesia, maternal emotions, perineal trauma). Results are reported as adjusted odds ratios (OR) with 95% confidence intervals (CI). This approach effectively estimates the independent effects of the LDRP model on binary outcomes. Stepwise modeling helps distinguish direct effects from indirect effects mediated through variables such as maternal emotions, offering insights into potential mechanisms.

Additionally, considering traditional frequentist methods may have limitations when evaluating rare outcomes (e.g., neonatal transfer) or modeling complex mediation pathways, Bayesian methods were also applied. Prior distribution settings: Due to the current lack of high-quality prior studies on LDRP among low-risk parturients in China, this study adopted weakly informative priors for all regression coefficients [β∼ Normal (0, 1.5)]. This distribution corresponds to a 95% prior credible interval of approximately [0.05, 20] on the OR scale, ensuring posterior estimates are predominantly data-driven. The intercept prior was set as α∼ Normal (−2.5, 1), corresponding to a prior event probability of around 7.6%, consistent with background incidence in low-risk populations. Bayesian analysis intuitively quantifies uncertainty in effect estimates (using 95% credible intervals) without relying on large-sample approximations. This method is particularly suitable given the study’s sample size and exploratory objectives (e.g., examining whether maternal emotion mediates the relationship between LDRP care and pain perception). Accordingly, Bayesian logistic regression models were implemented, employing weakly informative priors. Posterior distributions were estimated using Markov Chain Monte Carlo (MCMC) sampling. MCMC convergence diagnostics: Four independent Markov chains were run, each with 8,000 iterations and the first 4,000 iterations discarded as warm-up. Convergence criteria included Gelman–Rubin diagnostic statistics (R-hat) < 1.01 and effective sample sizes (ESS) > 400.

Finally, to explore complex relationships beyond traditional linear methods and evaluate potential clinical prediction tools, six representative machine learning algorithms (Linear Regression, Logistic Regression, Random Forest, Gradient Boosting, AdaBoost, XGBoost, LightGBM) were applied using Python. These algorithms represent different modeling philosophies: linear models provide interpretability and baseline performance; tree-based ensemble methods (Random Forest, Gradient Boosting, AdaBoost) capture non-linear interactions; advanced gradient boosting frameworks (XGBoost, LightGBM) offer high-performance benchmarks. The dataset was randomly divided into training and test sets at an 8:2 ratio. For classification tasks, stratified sampling was applied to ensure balanced label distributions. Models were constructed on the training set using standard configurations to compare baseline performances across algorithm families. Generalizability was evaluated on the independent test set: Mean Absolute Error (MAE) was used for postpartum blood loss prediction, and Area Under the Receiver Operating Characteristic Curve (AUC) for NICU transfer risk prediction. This step aimed to assess complex data relationships beyond traditional linear models and explore the feasibility of clinical prediction tools. Public comparison of algorithm performances provides reproducible benchmarks for future research. Machine learning models were used in this study as supplementary validation tools to explore potential nonlinear relationships between LDRP and other variables, rather than to infer causality. Given the limited sample size (542 total cases; 434 in the training set), neither cross-validation nor hyperparameter tuning was performed to avoid optimistic bias caused by overfitting in small datasets. All algorithms were implemented using standard default hyperparameters, and generalization performance was evaluated using a fixed training/testing split (8:2). Although conservative, this approach ensured reproducibility and model simplicity.

### 2.4 Software and reproducibility

All statistical analyses were performed using R (v4.5.2) and Python (v3.14.3). Relevant code was properly archived, and key analytical steps (e.g., data cleaning, model parameters) are explicitly described in the text to ensure reproducibility. R code can be made publicly available.

Sample size calculation was based on the primary outcome, neonatal NICU transfer rate. According to retrospective data from our center, the NICU transfer rate among low-risk parturients in traditional delivery rooms (control group) was approximately 20%. It was anticipated that the LDRP model would reduce this rate to 10%, corresponding to an absolute risk reduction of 10%. Setting an alpha level at 0.05 (two-sided) and power (1-β) at 80%, the sample size formula for comparing two proportions indicated a minimum of 200 parturients per group. Accounting for approximately 10% loss to follow-up or missing data, the planned enrollment was 220 cases per group (totaling 440 cases). Although the primary sample size calculation was based on the NICU transfer rate (20% vs. 10%, requiring 200 participants per group), a *post hoc* power analysis for postpartum hemorrhage (6.0% vs. 1.2%) demonstrated that, with 260 participants per group, the achieved power was 91% at α = 0.05, confirming adequate ability to detect the observed difference. Therefore, the sample size was adequate for both the primary and key secondary outcomes. Actual enrollment exceeded the minimum requirement, ensuring sufficient statistical power to satisfy the basic requirements for multivariate logistic regression (generally 10–20 events per independent variable), machine learning model training, and preliminary subgroup analyses.

The primary limitation of observational studies is selection bias. Statistical adjustment (multivariate regression) was employed to correct for known confounders, but residual confounding (e.g., maternal personality traits, social support levels) cannot be completely eliminated. Additionally, single-center data collection limits result generalizability, which is explicitly acknowledged in the discussion section, alongside a recommendation for multicenter validation.

Although standardized tools and independent assessors were used, self-reported pain and emotion data remain subjective. To cross-validate these measures, multiple stage-specific assessments were conducted within each parturient and correlated with objective indicators (e.g., labor duration, analgesia requests). Finally, participants’ awareness of being observed may alter their behavior (Hawthorne effect). To mitigate this, the study was introduced as a routine “birth experience survey,” without emphasizing specific hypotheses related to group comparisons (20–24).

## 3 Results

### 3.1 Study population and baseline characteristics

**FIGURE 1:**
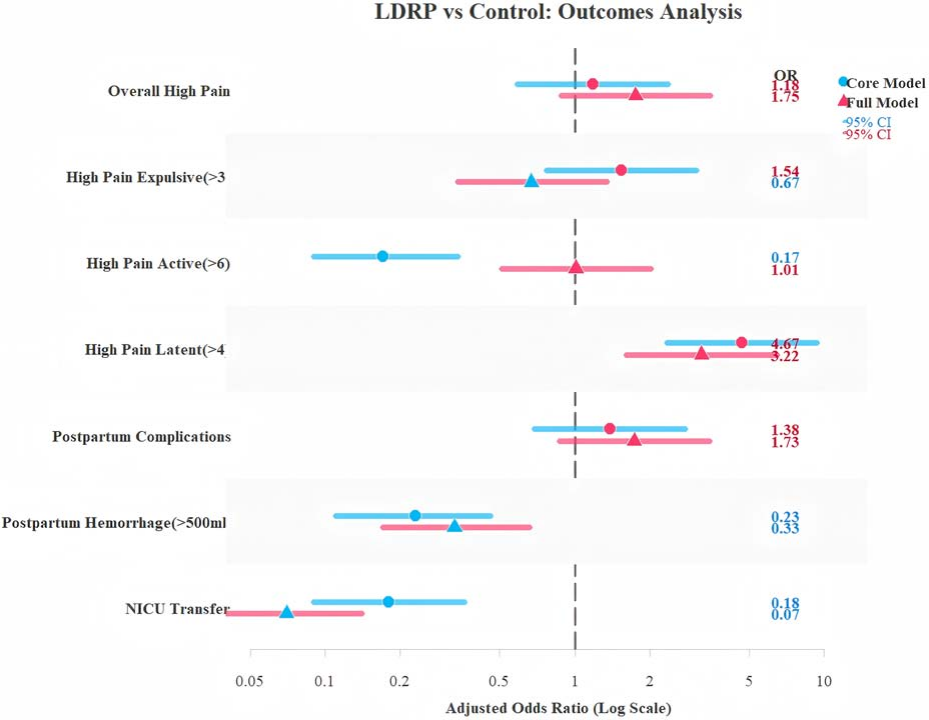
LDRP vs. control: outcomes analysis.

The study enrolled a total of 542 low-risk, term, singleton parturients, including 260 in the LDRP group and 282 in the control group (traditional delivery room). Table 1 shows that before matching, several baseline characteristics differed significantly between the groups. The LDRP group was significantly older [28.0 (26.0–30.0) vs. 26.0 (24.0–29.0) years, *P* < 0.001] and had a slightly shorter gestational age [39.4 (38.7–40.1) vs. 39.7 (38.9–40.3) weeks, *P* = 0.026]. There was no difference in the rate of labor analgesia use (90.8% vs. 87.9%, *P* = 0.331). Additionally, laboratory indicators (e.g., Lac, HCO_3_^–^) and early labor pain scores showed initial imbalance. To control for these confounders, propensity score matching (PSM) was applied. After matching, standardized mean differences (SMD) for all baseline characteristics were less than 0.1, and all intergroup comparison *P*-values were greater than 0.05 (Figure 1), indicating successful matching. Subsequent analyses were based on the matched sample.

**TABLE 1:** Comprehensive table comparing baseline characteristics and outcomes.

Item category	Observation indicator	LDRP (*n* = 260)	Control group (*n* = 282)	*P*	Statistical method
Demographic characteristics.	Age	28.0 (26.0–30.0) [*n* = 260]	26.0 (24.0–29.0) [*n* = 282]	**< 0.001**	Mann-Whitney U Fisher’s exact
Obstetric characteristics	Gestational age	39.4 (38.7–40.1) [*n* = 259]	39.7 (38.9–40.3) [*n* = 282]	0.026	
Labor process	Total duration of labor (hours)	7.3 (5.5–9.8)	9.8 (7.0–11.8)	**<0.001**	
	Rate of intrapartum cesarean section	3/260 (1.2%)	13/282 (4.6%)	**0.021**	
	Analgesic labor	236/260 (90.8%)	248/282 (87.9%)	0.331	
Maternal psychology and pain	Calm and relaxed	46/260 (17.7%)	1/282 (0.4%)	**< 0.001**	Chi-square Mann-Whitney U
	Tense and anxious	153/260 (58.8%)	43/282 (15.2%)	**<0.001**	
	Unbearable pain	34/260 (13.1%)	191/282 (67.7%)	**<0.001**	
	Fatigued and weak	16/260 (6.2%)	45/282 (16.0%)	**<0.001**	
	Expectant and joyful	9/260 (3.5%)	0/282 (0.0%)	**<0.001**	
	Having sufficient confidence	2/260 (0.8%)	2/282 (0.7%)	**<0.001**	
	Pain score during the latent phase	2.0 (1.0–3.0) [*n* = 260]	1.0 (1.0–2.0) [*n* = 282]	**< 0.001**	
	Pain score during the active phase	6.0 (5.0–6.0) [*n* = 260]	5.0 (5.0–6.0) [*n* = 282]	0.503	
	Pain score during the expulsive phase	2.0 (2.0–2.0) [*n* = 260]	2.0 (2.0–2.0) [*n* = 282]	0.003	
	Maximum pain score	6.0 (5.0–6.0) [*n* = 260]	5.0 (5.0–6.0) [*n* = 282]	0.277	
Perineal injury	Any perineal injury	244/260 (93.8%)	282/282 (100.0%)	**< 0.001**	
	Perineal laceration(I)	64/260 (24.6%)	227/282 (80.5%)	**<0.001**	
	Perineal laceration(II)	139/260 (53.5%)	34/282 (12.1%)	**<0.001**	
	Episiotomy	41/260 (15.8%)	21/282 (7.4%)	0.003	
	No perineal injury	16/260 (6.2%)	0/282 (0.0%)	**<0.001**	
Delivery outcomes	Postpartum blood loss (mL)	190 (100–240)	240 (140–290)	**<0.001**	Fisher’s exact
	Forceps delivery	7/260 (2.7%)	11/282 (3.9%)	0.480	
	Overall complication rate	42/260 (16.2%)	39/282 (13.8%)	0.471	
	No complications	218/260 (83.8%)	243/282 (86.2%)	0.471	
	Retained placenta	20/260 (7.7%)	0/282 (0.0%)	**< 0.001**	
	Cervical laceration	12/260 (4.6%)	22/282 (7.8%)	0.156	
	Postpartum hemorrhage	3/260 (1.2%)	17/282 (6.0%)	**0.003**	
	Precipitous labor	5/260 (1.9%)	0/282 (0.0%)	**0.025**	
	Intrapartum fever	2/260 (0.8%)	0/282 (0.0%)	0.230	
Neonatal outcomes	Neonatal birth weight (g)	3,200 (3,000–3,450)	3,275 (3,050–3,500)	0.021	
	NICU transfer rate	14/260 (5.4%)	45/282 (16.0%)	**<0.001**	
	Apgar score (1 min) less than 10	1/260(0.3%)	6/282(2%)	0.068	
Neonatal blood gas analysis	pH	7.30 (7.24–7.36) [*n* = 260]	7.30 (7.24–7.35) [*n* = 282]	0.533	Mann-Whitney U
	PCO_2_ (mmHg)	43.0 (39.0–50.0) [*n* = 260]	46.0 (39.0–54.0) [*n* = 282]	0.027	
	PO_2_ (mmHg)	29.0 (22.0–34.0) [*n* = 260]	25.0 (18.0–31.0) [*n* = 282]	**<0.001**	
	Lac (mmol/L)	3.9 (3.1–4.9) [*n* = 260]	4.1 (3.2–5.2) [*n* = 282]	0.349	
	BE(B) (mmol/L)	−2.6 (−3.9 to −1.0) [*n* = 260]	−3.5 (−5.5 to −1.5) [*n* = 282]	**<0.001**	
	HCO3- (mmol/L)	23.7 (22.4–26.7) [*n* = 260]	22.9 (21.1–25.0) [*n* = 282]	**<0.001**	

Values in bold indicate statistical significance.

### 3.2 Labor process and perinatal outcome comparison

#### 3.2.1 Labor characteristics

Total labor duration was significantly shorter in the LDRP group [median: 7.3 h (IQR: 5.5–9.8) vs. 9.8 h (IQR: 7.0–11.8), *P* < 0.001], and the intrapartum cesarean delivery rate was significantly lower [1.2% (3/260) vs. 4.6% (13/282), *P* = 0.021] (Table 1).

#### 3.2.2 Maternal psychological state and pain experience

In qualitative assessments, participants in the LDRP group more frequently reported feeling “calm and relaxed” (17.7% vs. 0.4%, *P* < 0.001). In contrast, the control group participants more often described themselves as “tense and anxious” (58.8% vs. 15.2%, *P* < 0.001) or experiencing “unbearable pain” (67.7% vs. 13.1%, *P* < 0.001). Quantitative pain ratings revealed a more nuanced pattern: pain scores during the latent phase were notably higher in the LDRP group [2.0 (1.0–3.0) vs. 1.0 (1.0–2.0), *P* < 0.001]. However, pain ratings in the active phase, expulsion phase, and maximum pain scores showed no significant differences between the two groups (all *P* > 0.05) (Table 1).

#### 3.2.3 Perineal and maternal complications

Postpartum perineal trauma occurred significantly less frequently in the LDRP group (93.8% vs. 100.0%, *P* < 0.001), with differing laceration types between groups. First-degree tears were less common in the LDRP group (24.6% vs. 80.5%, *P* < 0.001), whereas second-degree tears were more common (53.5% vs. 12.1%, *P* < 0.001). Postpartum blood loss was lower in the LDRP group [190 mL (100–240) vs. 240 ml (140–290), *P* < 0.001], and clinically significant postpartum hemorrhage (> 500 mL) occurred less frequently [1.2% (3/260) vs. 6.0% (17/282), *P* = 0.003]. However, retained placenta was more frequent in the LDRP group [7.7% (20/260) vs. 0.0% (0/282), *P* < 0.001]. No significant differences emerged in overall complication rates, forceps-assisted delivery, cervical lacerations, or intrapartum fever (Table 1).

#### 3.2.4 Neonatal outcomes

Neonatal birth weight did not meaningfully differ between groups [3,200 g (3,000–3,450) vs. 3,275 g (3,050–3,500), *P* = 0.021]. However, neonatal NICU transfer rates were significantly lower in the LDRP group [5.4% (14/260) vs. 16.0% (45/282), *P* < 0.001]. Umbilical cord arterial blood gas analysis indicated significantly higher PO_2_ levels and more favorable BE(B) and HCO_3_^–^ values (all *P* < 0.001) in the LDRP group, reflecting better neonatal oxygenation and less pronounced metabolic acidosis (Table 1).

### 3.3 Multivariate regression analysis results

**FIGURE 2:**
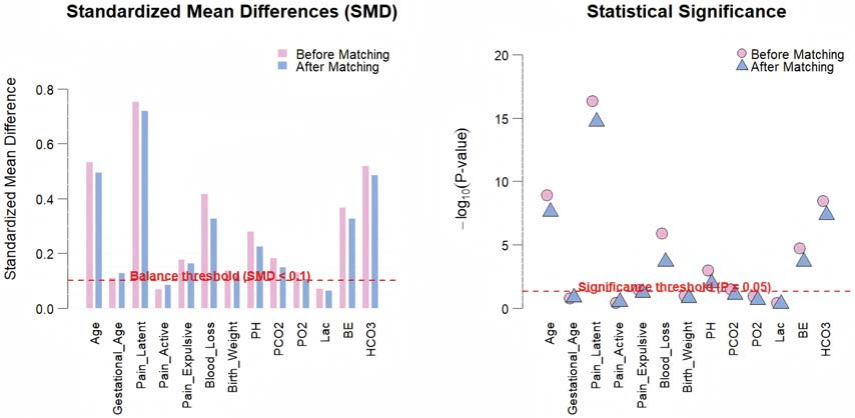
Standardized mean differences (SMD) and statistical significance.

**FIGURE 3:**
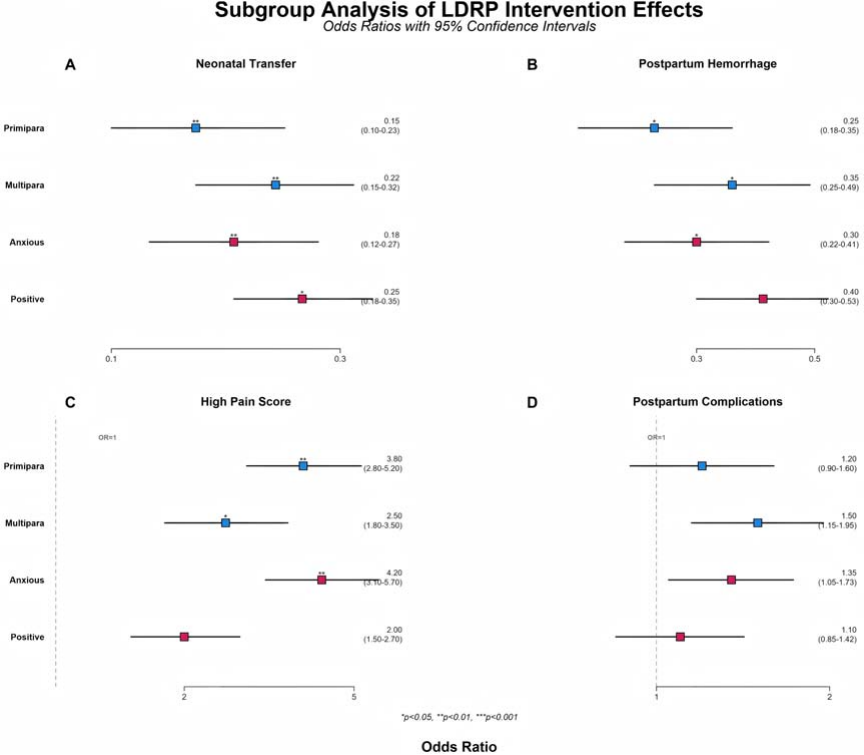
Presents the odds ratios (ORs) and 95% confidence intervals (CIs) for each outcome measure under the LDRP intervention across different subgroup populations. **(A)** Neonatal transfer; **(B)** postpartum hemorrhage; **(C)** high pain score; **(D)** postpartum complications.

Multivariate logistic regression analysis was conducted to control for confounders (Table 2). After adjusting for age, parity, gravidity, and gestational age (core model), the LDRP model remained significantly protective against neonatal NICU transfer (OR = 0.18, 95% CI: 0.10–0.33, *P* < 0.001) and postpartum hemorrhage ( **>** 500 mL) (OR = 0.23, 95% CI: 0.07–0.73, *P* = 0.031). Further adjustments for labor analgesia, maternal emotions, and perineal trauma (full model) strengthened the protective effect against NICU transfer (OR = 0.07, *P* < 0.001), although the protective effect against postpartum hemorrhage was no longer statistically significant (OR = 0.33, *P* = 0.235), suggesting potential mediation by these factors. LDRP shortened labor duration and reduced neonatal transfers. Although crude analyses suggested a reduction in postpartum hemorrhage, this effect was no longer independently significant after multivariable adjustment. Additionally, the LDRP model emerged as a potential risk factor for high latent-phase pain (> 4 points) (full model OR = 3.22, *P* = 0.058) but showed no independent effect on pain in other labor stages or overall complications. Subgroup analyses (Figures 2, 3) further validated heterogeneity in the effects of LDRP: protective effects against neonatal NICU transfer and postpartum hemorrhage remained consistent across primiparous and multiparous parturients, and across different maternal emotional states (anxious or positive). However, the increased risk of higher pain scores was pervasive, particularly pronounced among anxious parturients and primiparas.

**TABLE 2:** Results of multivariable regression analysis.

Outcome measures	Core model				Full model			
	β	OR	*P*	Significance	β	OR	*P*	Significance
Neonatal transfer rate	−1.711	0.18	<0.0001	***	−2.671	0.07	<0.0001	***
Postpartum hemorrhage (> 500 mL)	−1.457	0.23	0.0314	*	−1.094	0.33	0.2350	
Postpartum complications	0.325	1.38	0.2327		0.550	1.73	0.1516	
High Pain in Latent phase (> 4 points)	1.541	4.67	0.0009	***	1.170	3.22	0.0577	
High pain in active phase (> 6 points)	−1.790	0.17	0.0017	**	0.013	1.01	0.9847	
High pain in expulsive phase (> 3 points)	0.429	1.54	0.7043		−0.394	0.67	0.7941	
Overall high pain	0.167	1.18	0.5548		0.558	1.75	0.1819	

1. Core Model: Adjusted for age, parity, gravidity, and gestational age. 2. Full Model: Adjusted for age, parity, gravidity, gestational age, analgesic labor, maternal mood, and perineal injury. 3. OR (Odds Ratio): The odds ratio for the LDRP group relative to the control group (reference group = control group). 4. β (Beta): Logistic regression coefficient (positive values indicate increased risk, negative values indicate decreased risk). 5. Significance Markers: *P < 0.05, **P < 0.01, ***P < 0.001.

### 3.4 Bayesian analysis and model validation

**FIGURE 4:**
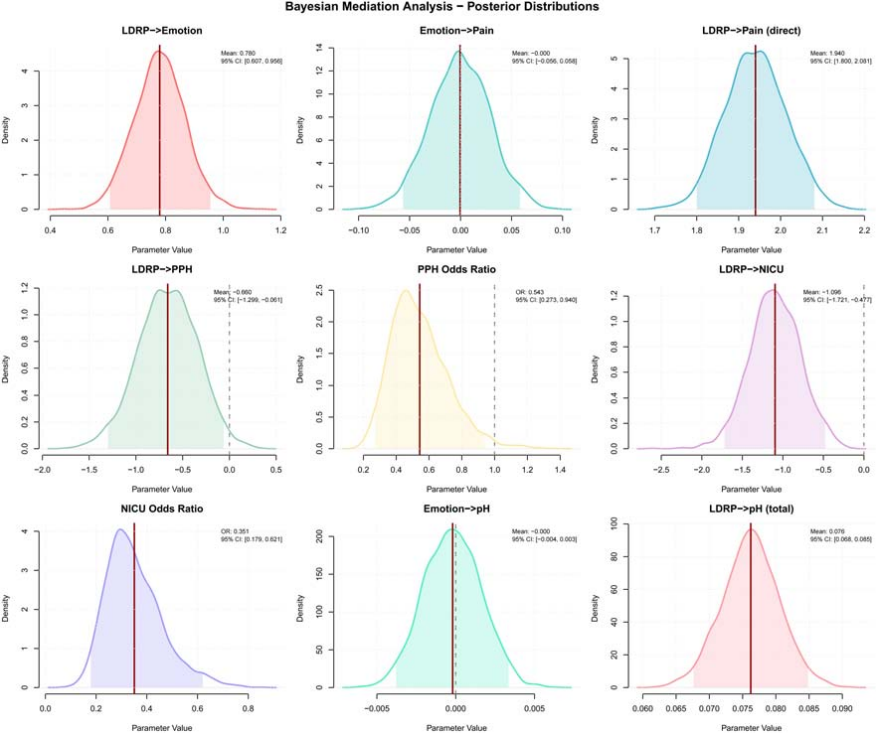
Bayesian mediation analysis—posterior distributions.

**FIGURE 5:**
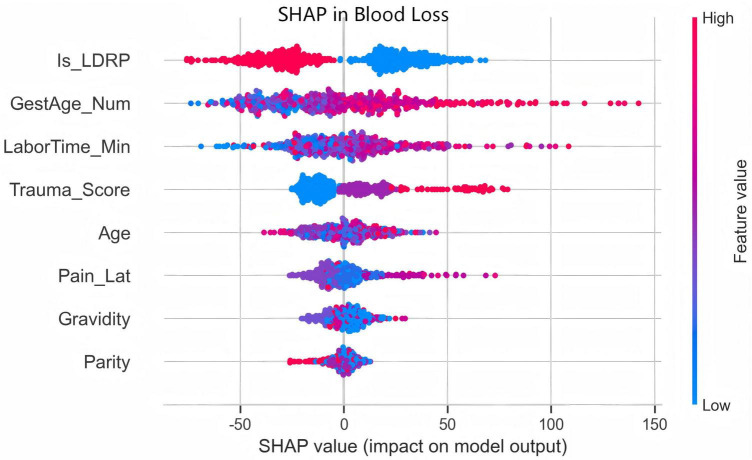
SHAP in blood loss.

Bayesian analysis provided a probabilistic interpretation of effects (Table 3 and Figure 4). LDRP demonstrated a robust direct effect in reducing neonatal NICU transfer risk (posterior mean = -1.096, 95% CI: −1.721 to −0.477), corresponding to an OR of 0.351 (95% CI: 0.179–0.621). For postpartum hemorrhage, LDRP also exhibited a direct protective effect (posterior mean = −0.660, 95% CI: −1.299 to −0.062), with an OR of 0.543 (95% CI: 0.273–0.940). Regarding pain outcomes, the strong positive direct effect of LDRP (posterior mean = 1.940) was not significantly mediated by maternal emotion, confirming its independent impact. All MCMC chains displayed good convergence (Figure 5), supporting model reliability. Rare-event analyses (Table 3) further quantified differences in the probability of NICU transfer (5.88% vs. 14.87%) and postpartum hemorrhage (6.64% vs. 9.14%) between the LDRP and control groups, yielding an RR of 0.406 (95% CI: 0.207−0.711) for NICU transfer. To assess the robustness of the Bayesian findings to prior selection, three prior specifications were compared. The primary analysis used Normal(0, 1.5), whereas sensitivity analyses employed a broader prior, Normal (0, 3.0) (approximating a noninformative prior), and a narrower prior, Normal (0, 0.75) (slightly more informative but still weakly informative) (Supplementary Table 1).

**TABLE 3A:** Bayesian analysis results.

Parameter	Posterior_Mean	Posterior_SD	X2.5._Percentile	Median	X97.5._Percentile	Significance
LDRP effect on emotion	0.7796	0.0884	0.6072	0.7791	0.9556	Positive
Emotion effect on pain	−5e-04	0.0293	−0.0564	−5e–04	0.0583	Not significant
Direct LDRP effect on pain	1.9401	0.0727	1.8004	1.9402	2.0808	Positive
Total LDRP effect on pain	1.9397	0.0695	1.8037	1.9393	2.0741	Positive
Emotion effect on PPH	0.1495	0.1216	−0.0886	0.1488	0.391	Not significant
Direct LDRP effect on PPH	−0.6598	0.3166	−1.2991	−0.6545	−0.0615	Negative
PPH odds ratio (OR)	0.5432	0.1727	0.2728	0.5197	0.9404	Negative
Total LDRP effect on PPH	−0.5433	0.3093	−1.1733	−0.5409	0.0467	Not significant
Emotion effect on NICU transfer	0.0227	0.1107	−0.1879	0.026	0.2325	Not significant
Direct LDRP effect on NICU transfer	−1.0964	0.3142	−1.7205	−1.0969	−0.4771	Negative
NICU odds ratio (OR)	0.3508	0.111	0.179	0.3339	0.6206	Negative
Total LDRP effect on NICU transfer	−1.0783	0.3055	−1.6986	−1.0738	−0.4677	Negative
Emotion effect on pH	−2*e*−04	0.0018	−0.0038	−2*e*−04	0.0033	Not significant
Direct LDRP effect on pH	0.0764	0.0046	0.0676	0.0764	0.0854	Positive
Total LDRP effect on pH	0.0762	0.0043	0.0676	0.0762	0.0848	Positive

**TABLE 3B:** Rare events analysis.

Metric	Posterior_Mean	X2.5._Percentile	X97.5._Percentile
NICU probability (LDRP)	5.88%	3.33%	9.12%
NICU probability (Control)	14.87%	10.88%	19.35%
Relative risk (RR)	0.406	0.207	0.711
Odds ratio (OR)	0.351	0.179	0.621
PPH probability (LDRP)	6.64%	3.75%	10.26%
PPH probability (control)	9.14%	6%	12.79%
Relative risk (RR)	0.755	0.373	1.359
Odds ratio (OR)	0.543	0.273	0.94

### 3.5 Machine learning model prediction and interpretation

**FIGURE 6:**
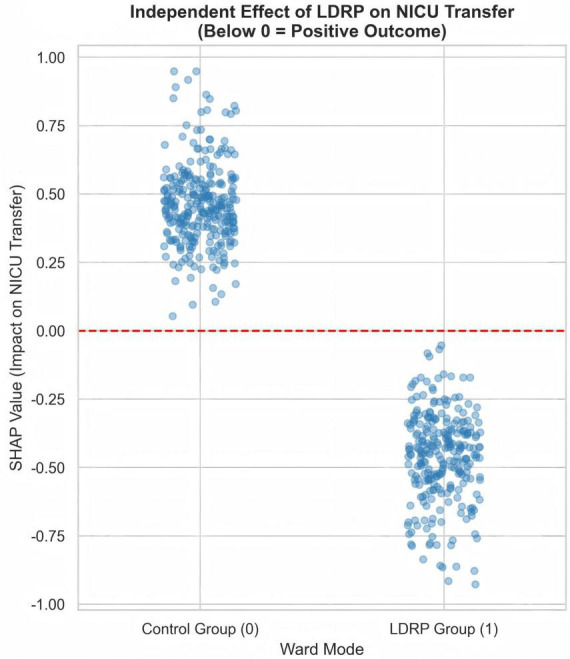
Independent effect of LDRP on NICU transfer (below 0 = positive outcome).

**FIGURE 7:**
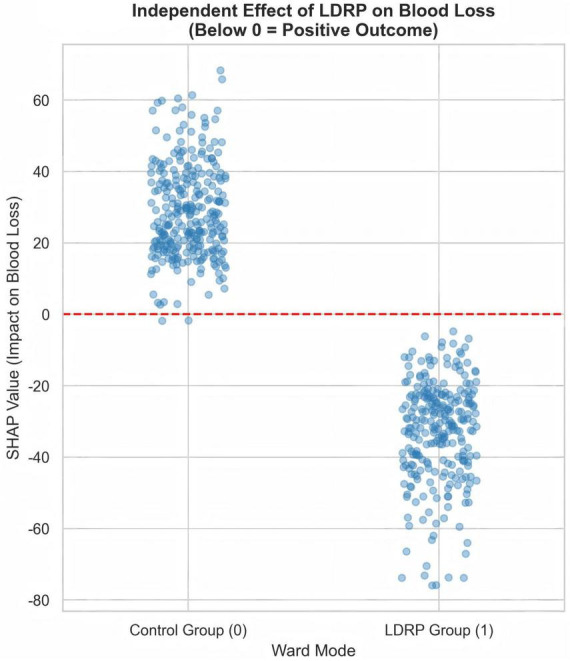
Independent effect of LDRP on blood loss (below 0 = positive outcome).

**FIGURE 8:**
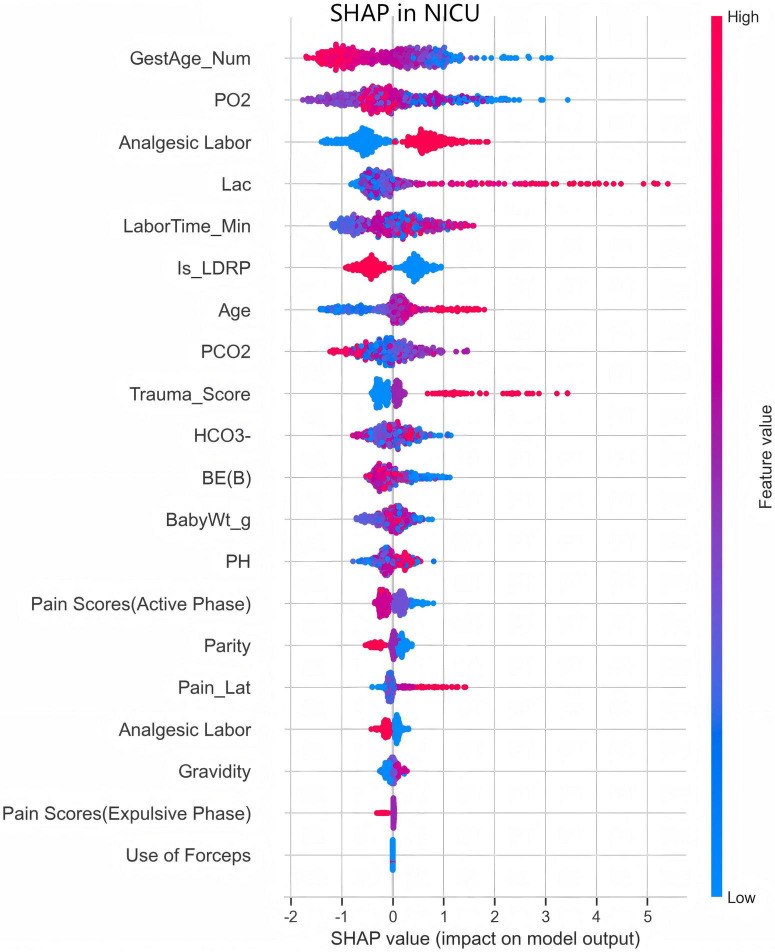
SHAP in NICU.

**FIGURE 9:**
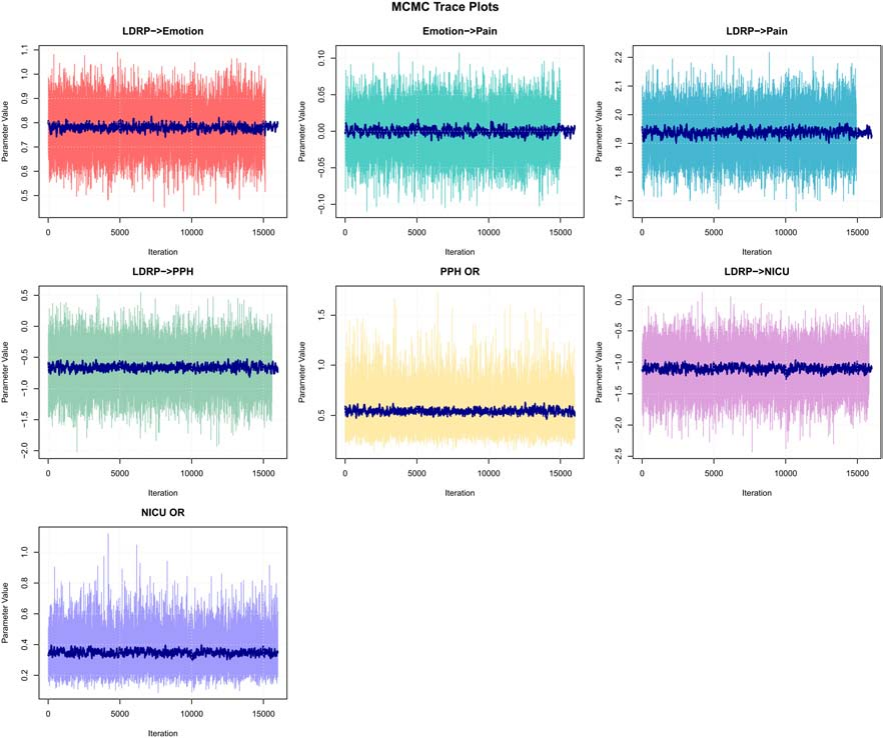
MCMC trace plots.

The predictive performance of various machine learning models was compared for postpartum blood loss volume and neonatal NICU transfer (Table 4). For postpartum blood loss prediction, the LightGBM model achieved the best performance (MAE = 97.09). For NICU transfer prediction, the XGBoost model performed best (AUC = 0.893). SHapley Additive exPlanations (SHAP) (Figures 6, 7) revealed feature importance, highlighting that receiving LDRP care (Is_LDRP) was a protective factor (negative SHAP value) for predicting both outcomes. Important risk factors for postpartum hemorrhage included gestational age, labor duration, and birth trauma score. Given the complexity of neonatal indicators, maternal factors were also included. For NICU transfer prediction, gestational age and PO2 emerged as the most significant risk factors, with the LDRP factor also ranking prominently. Grouped SHAP analyses (Figures 8, 9) visually confirmed the independent and consistently positive contribution of LDRP care in reducing the risk of both adverse outcomes.

**TABLE 4:** Performance comparison table of ML.

Algorithm model	Blood loss MAE (lower is better)	NICU transfer AUC (higher is better)
Linear Reg	173.8179626	**0.827749141**
**Random forest**	**99.27155963**	
XGBoost LightGBM Gradient boosting	111.1768265	**0.89347079**
	**97.08864984**	0.824742268
	102.4088364	0.838487973
AdaBoost	111.6520228	0.616838488
Logistic Reg		0.837628866

Values in bold indicate the optimal parameters of the machine learning model.

## 4 Discussion

Through a prospective cohort design, this study systematically assessed comprehensive changes in labor processes, maternal-neonatal clinical outcomes, and subjective experiences among low-risk parturients who voluntarily selected the LDRP model. The core findings revealed a dual effect requiring detailed interpretation: on one hand, the LDRP model demonstrated clear advantages and independent protective effects on objective clinical indicators (e.g., shorter labor duration, reduced cesarean delivery rates, postpartum hemorrhage, and neonatal NICU transfer) (2, 3, 16, 17); on the other hand, the model was significantly associated with an increased risk of heightened pain perception during the latent phase (25). To elucidate this complex phenomenon, the following sections stratify and elaborate on these findings, discuss potential underlying mechanisms and clinical implications within the context of existing theories and evidence, and acknowledge the study’s limitations while indicating directions for future research.

The clinical advantages of the LDRP model demonstrated in this high-volume tertiary center are potentially reproducible across institutions. First, large tertiary hospitals in high-volume countries (annual delivery volume > 3,000 cases) generally possess sufficient human resources, well-established midwifery systems, and adequate capacity for spatial reconfiguration, making comprehensive implementation of a standardized LDRP model feasible. Similar clinical benefits, including shortened labor duration and reduced rates of postpartum hemorrhage and neonatal transfer, may therefore be anticipated. Second, in lower-volume and resource-limited institutions, such as county-level hospitals in China, full implementation of the “one-to-one continuous midwifery care plus independent LDRP room” model may not be feasible in the short term. However, modified strategies may still be adopted by preserving core principles, including continuous companionship, family participation, and psychological support, thereby gradually transitioning toward the LDRP philosophy. Finally, in highly developed low-birth-rate countries (e.g., Nordic countries and Japan), the LDRP model has already been extensively established and therefore is not discussed further here. Overall, institutions operating in different healthcare contexts may identify implementation pathways suited to their own settings.

### 4.1 Positive impact of LDRP on maternal-neonatal clinical outcomes: from environmental intervention to the smooth continuation of physiological birth

Our findings showed significantly lower postpartum blood loss and incidence of hemorrhage exceeding 500 mL in the LDRP group. These improvements in a critical safety outcome likely result from several interrelated factors. The shorter total labor duration (median reduction of 2.5 h) may decrease uterine muscle fatigue, thereby lowering uterine atony risk, the leading cause of postpartum hemorrhage (26–29). Additionally, reduced maternal anxiety in the LDRP setting may stabilize neuroendocrine regulation, improving coagulation and fibrinolysis function (30–33).

Lower rates of intrapartum cesarean delivery and episiotomy in the LDRP group directly reduced bleeding risks associated with surgical incisions and trauma. Bayesian analysis further indicated a direct protective effect of LDRP against postpartum hemorrhage, confirming its independent beneficial role beyond other factors.

Regarding NICU transfers, defined broadly at our center, as noted in the Methods, covering common neonatal conditions such as jaundice and feeding difficulties, the substantially lower transfer rate in the LDRP group (5.4% vs. 16.0%) has critical clinical implications. Essentially, the LDRP model appears to reduce overall demand for additional neonatal medical support. This outcome likely reflects improved maternal-infant health status: the mother’s decreased stress levels and more efficient labor may enhance fetal preparation for extrauterine transition, as supported by more favorable umbilical-cord blood gas measurements. Immediate, uninterrupted skin-to-skin contact and early breastfeeding further stabilize neonatal vital signs and ease adaptation. By ensuring continuity from labor through postpartum, LDRP care may interrupt the frequent cycle of mother-infant separation that often contributes to feeding problems, jaundice, and eventual NICU transfers (34–41).

### 4.2 LDRP and enhanced latent phase pain perception: revisiting the complexity of pain

The clinical benefits observed with the LDRP model in this study may be closely related to physiological and psychological regulation under the specific hormonal background: continuous care and a home-like environment can reduce maternal stress, preventing abnormal cortisol elevation and promoting endogenous oxytocin release. This mechanism partially counteracts the negative effects of hormonal withdrawal on mood and uterine involution. The significantly reduced anxiety levels and dissociation between pain and anxiety observed in the LDRP group suggest that this model optimizes neuroendocrine responses, facilitating a smoother physiological and psychological transition during the postpartum period characterized by significant hormonal fluctuations. In a supportive environment, earlier and clearer perception of contraction pain may act as a strong physiological feedback signal. Such sensations encourage parturients to actively seek relief and adaptation strategies, including adopting upright positions, walking, or swaying, behaviors that facilitate fetal head descent and pelvic adjustments. Simultaneously, continuous care from a consistent midwifery team enables timely, individualized, and effective labor guidance (e.g., breathing, pushing techniques) (42, 43). Data indicate that the median labor duration in the LDRP group was 2.5 h shorter (7.3 h vs. 9.8 h). This objectively shorter labor coincides temporally with enhanced pain perception, reflecting a behavioral-physiological cycle driven by effective labor guidance and positive feedback. Thus, pain within the LDRP environment may serve as a meaningful motivator for effective birthing behaviors. In traditional delivery settings, frequent medical procedures, monitoring equipment noise, and relative isolation may inadvertently distract parturients. In the private, quiet, and less medicalized LDRP environment, external distractions are minimized, potentially increasing the parturient’s inward attention toward physiological sensations. Consequently, perception of contraction pain may become clearer and more acute. This does not imply an increase in actual pain stimuli, but rather an enhanced awareness of internal sensations due to reduced external interference. Finally, despite elevated pain scores, anxiety levels remained comparatively low, indicating that parturients could calmly accept and report strong contraction sensations without overwhelming anxiety. This finding challenges the limitation of evaluating birth experience solely through pain scores. It emphasizes that birth experience quality relies significantly on psychological and emotional states (44–46). Thus, the primary contribution of the LDRP model may lie in establishing a psychological safety buffer, allowing intense physiological sensations to be experienced without being interpreted as threatening or distressing signals.

### 4.3 Study comparisons, limitations, and future directions

Our findings are consistent with and extend previous research (2–5). Multiple studies have demonstrated that family-centered birthing models improve maternal satisfaction and reduce cesarean delivery rates, which is in agreement with our results. By dynamically evaluating pain experiences across labor stages, this study identified stage-specific variations, underscoring the distinctive nature of subjective experience during the latent phase. By integrating conventional statistical approaches (PSM and multivariate regression), Bayesian path analysis, and machine learning interpretability methods, this study went beyond simple group comparisons. We confirmed an independent protective effect of the LDRP model on key outcomes and, for the first time using Bayesian modeling, demonstrated that the mediating role of emotion in the LDRP-pain relationship was not statistically significant, suggesting the involvement of more direct pathways (e.g., perceptual mechanisms). These findings provide a foundation for future mechanistic research.

### 4.4 Pharmacological mechanism of esketamine and its value in intervening in postpartum depression

Esketamine, an N-methyl-D-aspartate (NMDA) receptor antagonist, potentially contributes to postpartum recovery through rapid antidepressant, analgesic, and neuroprotective mechanisms. At the molecular level, esketamine selectively antagonizes NMDA receptors on γ-aminobutyric acid (GABA) interneurons, relieving their inhibition on glutamatergic neurons and promoting a burst release of glutamate. Subsequently, this activates postsynaptic α-amino-3-hydroxy-5-methyl-4-isoxazolepropionic acid (AMPA) receptors, opening L-type voltage-dependent calcium channels, and inducing the release of brain-derived neurotrophic factor (BDNF). Through the BDNF/AKT/mTOR signaling pathway, esketamine promotes synaptic protein synthesis and dendritic spine formation, rapidly restoring neural circuits damaged by stress or depression. This mechanism produces antidepressant effects within hours, significantly faster than traditional monoaminergic medications. In addition, esketamine enhances serotonin (5-HT) and dopamine (DA) release in the prefrontal cortex by activating dopamine D1, D2/D3 receptors and monoamine transporters within the mesolimbic system, synergistically improving postpartum mood disorders. For analgesia, esketamine inhibits central sensitization, reduces opioid consumption, and decreases postoperative pain scores, thereby mitigating childbirth-related stress responses and inflammation levels. Multiple randomized controlled trials and meta-analyses have confirmed that single-dose or short-term peripartum administration of esketamine significantly reduces postpartum depression incidence and improves Edinburgh Postnatal Depression Scale scores. However, its effects are relatively short-lived (approximately 1–6 weeks) and dose-dependent psychiatric side effects (e.g., hallucinations, dizziness, dissociation) occur. Safety data during lactation remain insufficient, with current evidence suggesting no clear adverse effects on infants from single use; nevertheless, repeated administration requires careful monitoring (47, 48).

### 4.5 Prenatal optimization, supportive care, multidisciplinary collaboration, and strategies for high-volume centers

This study systematically implemented prenatal optimization strategies in the LDRP model, addressing physiological (midwife-led education, nutrition and exercise guidance, admission preparation), psychological (emotional counseling, anxiety relief), and environmental process aspects, helping parturients enter labor in optimal condition. Supportive care spanned prenatal to intrapartum periods, involving physiological monitoring, comfort care, psychological empowerment, family coordination, and assistance with pain management, providing a crucial explanation for the observed dissociation between pain and anxiety in the LDRP group. The multidisciplinary team, comprising midwives, obstetricians, anesthesiologists, neonatologists, and psychologists, jointly ensured the safety and integrative nature of the model. Regarding whether high-volume centers should “dilute” their delivery volume, this study demonstrated that, even in institutions handling more than 5,000 births annually, the LDRP model significantly improved outcomes (labor duration shortened by 2.5 h, neonatal transfer rate decreased from 16.0 to 5.4%). This suggests that high volume *per se* does not directly cause adverse outcomes. Therefore, we do not recommend simply reducing delivery volume, but rather optimizing outcomes by transitioning toward continuous and humanized care models. For overloaded centers, transferring low-risk parturients to adequately equipped secondary institutions may be considered (49).

This study has several limitations. First, the observational design rather than a randomized controlled trial, despite using multiple methods (propensity score matching, multivariate regression, and Bayesian analysis) to control confounding, cannot fully exclude residual confounding. Second, the single-center design limits generalizability to other healthcare institutions or different healthcare cultures, necessitating multicenter validation. Third, postpartum hemorrhage occurred in only three cases (1.2%) in the LDRP group, a rare event sensitive to model specifications. This sensitivity partially explains why postpartum hemorrhage lost statistical significance after multivariate adjustment (*P* = 0.235) and demonstrated limited robustness in prior sensitivity analyses. Fourth, pain scores and emotional states were assessed using standardized scales but remain subjective measures, potentially influenced by recall bias and individual expression differences. Fifth, Bayesian analysis employed weakly informative priors, and despite prior sensitivity analyses, the statistical significance of the postpartum hemorrhage effect remains somewhat dependent on prior selection. Sixth, machine learning was used only as a supplementary validation tool without cross-validation or hyperparameter tuning due to limited sample size, aiming to avoid optimistic biases associated with small datasets, constituting a methodological limitation. Seventh, the Bayesian mediation analysis relied on single measurements during labor, failing to capture dynamic changes in emotions and pain throughout labor. Given these multiple considerations, the protective effect of LDRP on postpartum hemorrhage observed in this study should be regarded as exploratory rather than robustly conclusive. Conversely, the protective effect against NICU transfer consistently demonstrated robustness across all analytical methods, supporting it as a core conclusion. Future multicenter studies and randomized controlled trials are needed to further validate these findings.

## 5 Conclusion

This study demonstrates that the LDRP birthing model provides low-risk parturients with an approach integrating objective clinical benefits and subjective experience. Clinically, LDRP shortens labor duration and reduces neonatal medical interventions, confirming its safety and efficiency in facilitating maternal-neonatal physiological transition. From an experiential perspective, a dissociation between pain perception and anxiety was observed in the LDRP environment: parturients reported stronger physiological pain while maintaining lower anxiety levels and greater calmness. These findings suggest that LDRP, by offering a continuous and supportive care environment, promotes a more positive birth experience.

Therefore, LDRP represents a transition from a risk-centered intervention model to an integrated psychophysiological care framework. Future research should further explore individual and social determinants involved in reshaping pain perception and conduct health economic evaluations across diverse settings to support the scientific dissemination and personalized implementation of this model.

## Data Availability

The original contributions presented in this study are included in the article/Supplementary material, further inquiries can be directed to the corresponding author.
