# Supplementary material for: The impact of the integrated labor-delivery-recovery-postpartum unit on maternal–neonatal outcomes and psychological experiences among low-risk parturients: a prospective cohort study from a high-volume tertiary center in china
Source: Front Med (Lausanne). 2026 Jun 22;13:1845643. doi: 10.3389/fmed.2026.1845643 (PMC13333597; doi:10.3389/fmed.2026.1845643)
Supplement: Supplementary file 1 [file Table_1.doc]

| **Outcome** | **Prior Setting** | **OR (95% CI)** | **95% CI excludes 1** | **R-hat** | **ESS** |
| --- | --- | --- | --- | --- | --- |
| NICU transfer | SD=1.5 (Primary analysis) | 0.333 (0.168-0.572) | Yes | 1.000 | 5352 |
| NICU transfer | SD=3.0 (Wide prior) | 0.318 (0.154-0.560) | Yes | 1.000 | 4950 |
| NICU transfer | SD=0.75 (Narrow prior) | 0.374 (0.200-0.633) | Yes | 1.000 | 5107 |
| Postpartum hemorrhage | SD=1.5 (Primary analysis) | 0.401 (0.026-0.618) | Yes | 1.000 | 5864 |
| Postpartum hemorrhage | SD=3.0 (Wide prior) | 0.203 (0.001-0.988) | Yes | 1.001 | 5685 |
| Postpartum hemorrhage | SD=0.75 (Narrow prior) | 0.691 (0.155-1.924) | No | 1.000 | 6536 |

****Note****: All models had R-hat < 1.01 and ESS > 4000.

S1：Results of the sensitivity analysis for the internal standard deviation of the prior distribution.
